# Optimal Design and Application of Universal Cementitious Material Prepared Using Full Industrial Solid Wastes

**DOI:** 10.3390/ma18153485

**Published:** 2025-07-25

**Authors:** Zilu Xie, Zengzhen Qian, Xianlong Lu, Bing Yue, Wendi Su, Mengze Tian

**Affiliations:** 1 School of Engineering and Technology, China University of Geosciences Beijing, Beijing 100089, China; 3002220008@email.cugb.edu.cn (Z.X.); yue@email.cugb.edu.cn (B.Y.); 2102240115@email.cugb.edu.cn (W.S.); 2102240116@email.cugb.edu.cn (M.T.); 2 State Grid Electric Power Engineering Research Institute Co., Ltd., Beijing 100032, China; luxianlong@163.com

**Keywords:** industrial solid wastes, cementitious materials, aeolian sand, stabilization, unconfined compressive strength, microstructure

## Abstract

This study developed a full solid waste-based cementitious material (ISWs-CM) using steel slag (SS), ground granulated blast furnace slag (GGBFS), phosphorus slag (PS), carbide slag (CS), and desulfurized gypsum (DG) to completely replace cement. A two-layer optimization strategy, combining three chemical moduli and simplex lattice experiments, was employed to determine the proportion and to investigate the impact of proportions on the uniaxial compressive strength of mortar. As an application case, the ISWs-CM with the optimal proportion was employed to stabilize aeolian sand, and its effectiveness as a cement substitute and the underlying mechanisms were investigated. The results indicated that the ISW proportion that maximized the strength of the mortar was SS:GGBFS:PS:CS = 5:20:20:40. The strength of the mortar was enhanced when the proportion of GGBFS exhibiting the highest reactivity was increased and also increased initially and then decreased with an increase in CS when the dosage of GGBFS was fixed. The aeolian sand stabilized by ISW-CM exhibited higher strength than that stabilized with cement. The greater number and variety of hydration products resulted in denser connections and encapsulation of sand particles, which highlights the synergistic effect of ISWs and the potential of ISW-CM as a cement replacement across diverse applications including aeolian sand stabilization.

## 1. Introduction

Cementitious stabilization or solidification is a ground improvement approach used worldwide for different types of soils. Owing to its wide availability, effectiveness, and easily controlled composition, ordinary Portland cement (OPC) has been commonly used to stabilize soils through in situ mixing and grouting, which deliver OPC additives as either powder or slurry to mix with the host soils [1,2,3,4,5]. However, environmental protection has become a major global concern, especially given the carbon peaking and carbon neutrality goals developed in recent years. The aforementioned soil stabilization technique using OPC does not satisfy the requirements of ecological construction because of the large CO_2_ emissions and high energy consumption that occur during OPC production [6,7,8].

On the other hand, large amounts of industrial solid waste (ISWs) have been rapidly generated with the development of the global economy, especially in regions with fast industrial development. Various ISWs have posed significant environmental risks due to their potential to pollute and even disrupt ecosystems [9,10,11,12,13,14,15]. However, the treatment of ISWs is extremely challenging, not only in terms of cost but also environmental protection owing to the unavoidably complex compositions and high impurity contents of ISWs.

In response to the aforementioned dual challenges of reducing CO_2_ emissions from OPC production and managing ISWs in an effective and efficient way, quite a few attempts have been made to explore alternative ISW-based cementitious materials to act as complete or partial substitutes for OPC in soil stabilization, such as fly ash and steel slag [16,17,18], magnesia–coal slag [19], ground granulated blast furnace slag [20,21], gypsum-based wastes [22,23], bauxite residue (red mud) [24,25,26,27,28], and mine tailings [29,30]. In general, the potential for a material to be useful as a replacement for cement mainly depends on the chemical and physical properties of the ISW-based cementitious material, which can be categorized into three different types of hydraulic properties, pozzolanic properties, and reactivity. However, because ISWs have great variability in their physical characteristics and chemical compositions, the current practice of soil stabilization using ISW-based cementitious materials instead of cement is generally fragmental. In existing studies using ISWs as OPC replacements for soil stabilization, trial-and-error experiments and the direct adoption of empirical values represent the most prevalent methods for choosing the proportions of cementitious materials [31,32,33,34,35,36]. However, these approaches result in poor development efficiency and render the determination of mix proportions devoid of a theoretical foundation. Some studies have reported the application of efficient experimental design methodologies, such as the Taguchi method, to investigate the proportions of solid wastes and their interactive effects on the strength-contributing effects in stabilized soil [37,38,39,40,41,42]. Nevertheless, most studies have treated ISWs merely as additives for partially replacing cement when developing cementitious systems, and the dosages of materials were assumed to be independently variable, thereby failing to account for the fundamental fixed-sum constraint that requires the total proportion of all materials to equal 100%. There are still great challenges in the synergistic preparation of cementitious binders using full industrial solid wastes.

It is noteworthy that, owing to the inconsistencies in the chemical compositions of the raw materials, it is difficult to achieve an appropriate raw mix proportion of OPC clinkers. To obtain the desired quality of the cement clinker with higher and optimal hydration and pozzolanic reactivity, the mix proportions of the raw materials are traditionally adjusted by referencing three chemical moduli (TCM), including the alumina modulus (IM), silica modulus (SM), and lime saturation coefficient (KH) [43,44,45,46,47]. As is well known, OPC’s TCM are based on the four main oxides—calcium oxide (CaO), silicon oxide (SiO_2_), aluminum oxide (Al_2_O_3_) and iron oxide (Fe_2_O_3_)—though the chemical components of OPC clinkers are represented as various oxides. Nowadays, the aforementioned synergistic theory, which has been successfully introduced to optimize the complementation of chemical constituents in different raw materials, has become a typical paradigm in the domain of cement production. Recently, the TCM approach, originally developed for OPC production, has also been employed as an acceptable method to guide the constituent design of ISW-based cementitious materials for expansive soil and coastal soft clay stabilization [3,17,18].

It is well known that aeolian sands are mainly present in desert areas, such as Inner Mongolia, Ningxia, Xinjiang, and Gansu Province in Northwest China [48,49]. However, aeolian sands are very peculiar materials due to their properties of extremely fine grain size and homogeneously poor grading, with a rounded shape and negligible plasticity [50], which results in difficulties in the construction of infrastructure. Despite the fact that there has been notable progress in evaluating the stabilization efficiency and potential of different stabilizers over the past few decades [50], OPC-based materials are still the main stabilizer for aeolian sands worldwide. Existing studies indicate that the utilization of OPC can effectively reduce the high natural porosity of aeolian sand through the formation of calcium silicate hydrate (C-S-H) and abundant crystalline compounds filling the pore spaces. Consequently, stabilized aeolian sand achieves a UCS exceeding 1.5 MPa and a cohesion greater than 150 kPa, with strength exhibiting exponential growth as the cement content increases [51,52,53]. Further research demonstrates that the strength of stabilized aeolian sand reaches its peak when the water content is 9% [49]. Regarding the stabilization mechanism, it is widely acknowledged that, unlike clays containing significant proportions of clay and silt particles, aeolian sand comprises over 98% sand-sized particles [50]. Furthermore, the SiO_2_ within aeolian sand particles is highly crystalline and does not undergo hydration under typical conditions [54,55,56]. Therefore, the strength of stabilized aeolian sand primarily originates from the gel phase and crystalline compounds synthesized by the cementitious materials.

In a previous study [44], a stabilizer suitable for aeolian sand was developed, demonstrating stabilization effects comparable to those stabilized with OPC. However, as this study specifically targeted aeolian sand—a soil with highly distinctive engineering properties—the resulting proportion may lack sufficient universality. Its application as a binder in concrete or for stabilizing other special soils remains challenging. Furthermore, as the essential precursor for the formation of ettringite (a primary hydration product in cement), calcium sulfate dihydrate (CaSO_4_·2H_2_O, gypsum) was absent in prior studies, which may result in the developed cementitious material struggling to match the performance of conventional cement when applied in other fields.

Based on the aforementioned previous study, this paper developed a more universal ISWs-CM by preparing mortars using five types of ISWs—steel slag (SS), ground granulated blast furnace slag (GGBFS), phosphorus slag (PS), carbide slag (CS), and supplementary desulfurized gypsum (DG) at 15 wt%—combined with standard sand. A dual-layer optimization strategy combining TCM theory and four-factor simplex lattice design was employed to determine the proportions of ISWs-CM, and the interactive effects of ISW dosage on the unconfined compressive strength (UCS) of mortars were investigated. A regression model was established to relate the ISW proportion to mortar UCS, and an analysis of variance (ANOVA) was employed to examine the sensitivity of mortar strength to variations in the ISW proportions and the interactions between different ISWs. Finally, the developed ISWs-CM was reapplied to stabilize aeolian sand, and its performance was compared with previous research findings. Additional microstructural characterization tests were conducted to elucidate the fundamental mechanism enabling the developed material to serve as a viable substitute for OPC. The results of this study demonstrated the potential of the developed ISWs-CM to replace cement across diverse applications including aeolian sand stabilization.

## 2. Methodological Framework

**Figure 1:**
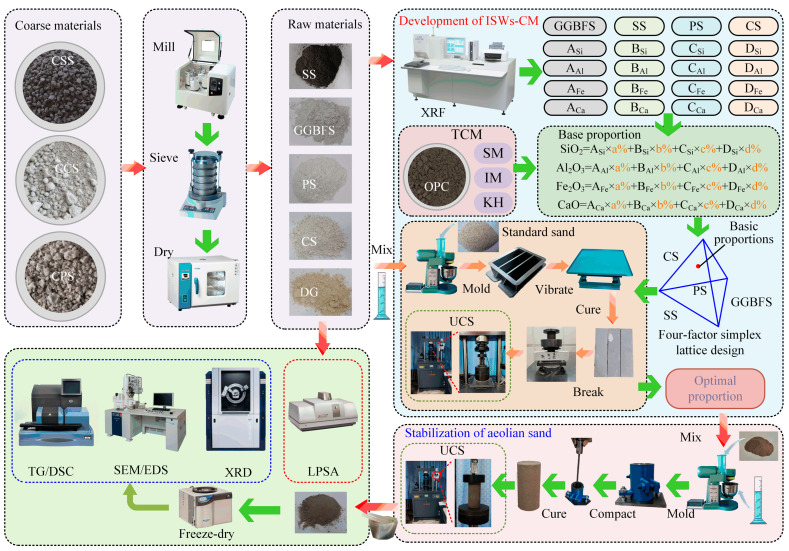
Process of ISWs-CM preparation and its application in aeolian sand stabilization.

The main purpose of this study is to develop a full ISW-based cementitious material to completely replace OPC for aeolian sand stabilization in desert regions based on a dual-layer optimization strategy combining TCM theory and four-factor simplex lattice design. The implementation proceeded in six steps:

(1) Based on the primary oxide composition (CaO, SiO_2_, Fe_2_O_3_, Al_2_O_3_) of OPC, four types of ISWs prevalent in China with substantial stockpile volumes were selected as the primary constituents for the ISW-based CM: SS, GGBFS, PS, and CS. These ISWs possess oxide compositions analogous to OPC and exhibit mutually complementary and adjustable proportions. Specifically, SS, GGBFS, and PS were utilized to provide hydraulically active minerals such as C_2_S and C_3_S for hydration reactions in appropriate proportions, while CS was employed to regulate the alkalinity of the system. Additionally, DG was incorporated to enhance the compactness of the system.

(2) The selected raw ISWs were ground to a fineness similar to OPC, and the contents of the major oxides (i.e., CaO, Al_2_O_3_, SiO_2_ and Fe_2_O_3_) of OPC and each ISW were determined.

(3) The basic proportions of each ISW of SS, GGBFS, PS, and CS were obtained (except an initial gypsum content of 15 wt%) to thus prepare a mixture with the same TCM as the referenced OPC clinker, and these were calculated using the proposed dosage of each ISW and its corresponding oxide composition content.

(4) A total of 25 mortar specimens were produced by mixing the standard sand and ISWs, in which the dosages of SS, GGBFS, PS, and CS were determined by the four-factor simplex lattice method within a 10% range centered on the basic proportion, and the dosage of DG was fixed as 15%.

(5) ISWs-CM with the optimal proportion of each ISW was developed by conducting UCS tests of the aforementioned 25 mortar specimens and analyzing the influence of each ISW dosage on UCS.

(6) The ISWs-CM developed to stabilize aeolian sand were employed, and a comparative analysis of the phase composition and microstructure was conducted between aeolian sand stabilized with the ISWs-CM developed in this study (ASISW), the ISWs-CM developed in a previous study [44], and the OPC (ASOPC) reported on in the literature to elucidate the fundamental mechanism enabling the developed ISW-CM to serve as a viable substitute for OPC. The methodological framework of this study is shown in Figure 1.

## 3. Materials and Experimental Procedures

### 3.1. Materials

The GGBFS used in this study is S95-grade granulated blast furnace slag that complies with the specifications outlined in the Chinese GGBFS standard (GB/T 18046, 2017) [57]. It is formed during the blast furnace ironmaking process from the gangue present in iron ore, the ash content in fuel, and the non-volatile components of limestone. It has now found relatively widespread application in the development of novel cementitious materials.

Coarse steel slag (CSS), phosphorus slag (CPS), and carbide slag (CCS) are generated during steelmaking, yellow phosphorus production via the electric furnace process, and acetylene production using calcium carbide, respectively. Currently, the predominant disposal method for these materials is stockpiling, with limited further resource utilization efforts. In this study, these coarse materials were collected from a steel mill, a phosphorus chemical plant, a calcium carbide plant, and an acetylene gas plant located in northern China. In the laboratory, the coarse materials were individually ground using a planetary ball mill at a ball-to-material ratio of 1:1 and a rotational speed of 520 rpm for 90 min and sieved to pass through a 200-mesh sieve (75 μm).

DG is produced by the absorption of SO_2_ using CaO in coal-fired power plants. Research on utilizing DG as a substitute for natural gypsum has already been conducted.

**Figure 2:**
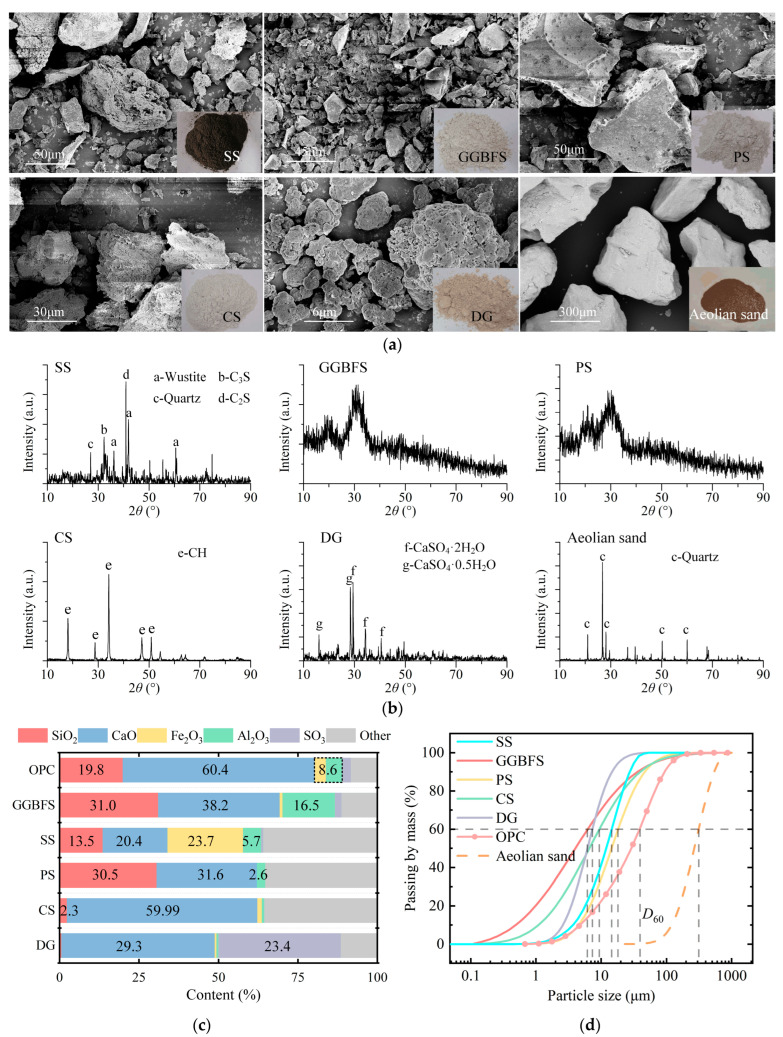
Raw material properties: (**a**) particle morphologies, (**b**) mineral phase composition, (**c**) chemical composition, and (**d**) particle size distribution.

The microscopic particle morphologies of materials observed using scanning electron microscopy (SEM, SU8220, HITACHI High-tech Co., Ibaraki, Japan), mineral phase composition measured by X-ray diffraction (XRD, D8-Advance, Bruker, Hongkong, China), chemical compositions determined using an XRF 1800 wavelength dispersive X-ray fluorescence spectrometer (Shimadzu, Kyoto, Japan), and particle size distributions measured using a Mastersizer 2000 laser particle size analyzer (LPSA) (Malvern Ins., Worcester, UK) are shown in Figure 2.

The standard sand used meets the requirements specified by the ISO standard (ISO-679, 2020) [58] and was produced by the ISO Standard Sand Co. Ltd., Xiamen, China.

Aeolian sand was collected from Taklimakan Desert in Xinjiang, China, at a depth of over 1.5 m below the ground surface. All samples were immediately placed in sealed bags and stored in a dark environment after excavation. Figure 2a illustrates the macroscopic morphology, microscopic particle morphology, and particle size distributions of the aeolian sand. The particles exhibit good roundness, with surfaces featuring corrosion pits and secondary precipitates. The *D*_60_ of aeolian sand is 345.5 μm, and the coefficient of uniformity is 2.62, classifying it as poorly graded sand (SP) according to the American standard (ASTM D2487, 2025) [59].

### 3.2. Specimen Design

#### 3.2.1. Development of ISWs-CM Based on Two-Layer Optimization Strategy

For the development of ISWs-CM, the oxide content of P.O. 42.5 ordinary Portland cement produced by China United Cement Corporation was used as a reference for the proportioning of ISWs.

TCM theory posits that the engineering properties of cement clinker, including strength and workability properties (e.g., fluidity and setting time), are predominantly governed by the mass ratio of four principal oxides: CaO, SiO_2_, Al_2_O_3_, and Fe_2_O_3_. As defined in Equations (1)–(3), the parameters of TCM include the following: SM, calculated by the mass percentage ratio of SiO_2_ to the sum of Al_2_O_3_ and Fe_2_O_3_, representing the relative contents of C_2_S, C_3_S, tricalcium aluminate (C_3_A), and tetracalcium aluminoferrite (C_4_AF), which govern the proportion between silicate minerals and flux minerals in the clinker and thereby determine the relative contents of C_2_S and C_3_S; IM, indicating the mass percentage ratio of Al_2_O_3_ to Fe_2_O_3_, which determines the ratio between C_3_A and C_4_AF in the clinker; and KH, characterizing the degree of SiO_2_ saturation by CaO to form C_3_S, consequently determining the presence of free CaO [18]. Based on Equations (1)–(3), the calculated TCM parameters for the reference cement standard are SM = 2.29; IM = 1.47; and KH = 0.91, which fall within conventional acceptable ranges.

(1) $$SM=\frac{C_{3}S+1.325C_{2}S}{1.434C_{3}A+2.046C_{4}\mathrm{AF}}=\frac{{\mathrm{SiO}}_{2}}{{\mathrm{Al}}_{2}O_{3}+{\mathrm{Fe}}_{2}O_{3}},\;(1.7\leq \mathrm{SM}\leq 2.7)$$

(2) $$IM=\frac{1.15C_{3}A}{C_{4}\mathrm{AF}}=\frac{{\mathrm{Al}}_{2}O_{3}}{{\mathrm{Fe}}_{2}O_{3}},\;(0.9\leq \mathrm{IM}\leq 1.7)$$

(3) $$KH=\frac{C_{3}S+0.88C_{2}S}{C_{3}S+1.33C_{2}S}=\frac{\mathrm{CaO}-1.65{\mathrm{Al}}_{2}O_{3}-0.35{\mathrm{Fe}}_{2}O_{3}}{2.8{\mathrm{SiO}}_{2}},\;(0.9\leq \mathrm{KH}\leq 1.0)$$

Determining ISW proportions involves adjusting the dosages of individual ISWs to ensure the mass ratio of the oxides of the final mixture aligns with the TCM of the reference cement. Therefore, a system of linear equations shown in Equation (4) was established based on the contents of the four oxides in the constituent ISWs. Herein, the left-hand-side terms represent the total content of a specific oxide derived from the blended ISWs (calculated as the oxide content in each ISW multiplied by its proportion), while the right-hand-side terms denote the target oxide concentrations required to satisfy the cement clinker’s TCM. Solving Equation (4) yields the following reference proportion: SS:GGBFS:PS:CS = 6.6:12.3:23.5:42.7.

(4) $$\left\{\begin{array}{l} 13.58\mathrm{SS}+31.07\mathrm{GGBFS}+30.52\mathrm{PS}+2.31\mathrm{CS}=19.87\\ 20.40\mathrm{SS}+38.22\mathrm{GGBFS}+31.60\mathrm{PS}+59.99\mathrm{CS}=60.45\\ 23.78\mathrm{SS}+0.88\mathrm{GGBFS}+0.05\mathrm{PS}+1.37\mathrm{CS}=3.50\\ 5.73\mathrm{SS}+16.5\mathrm{GGBFS}+2.61\mathrm{PS}+0.75\mathrm{CS}=5.17 \end{array}\right.$$

**Figure 3:**
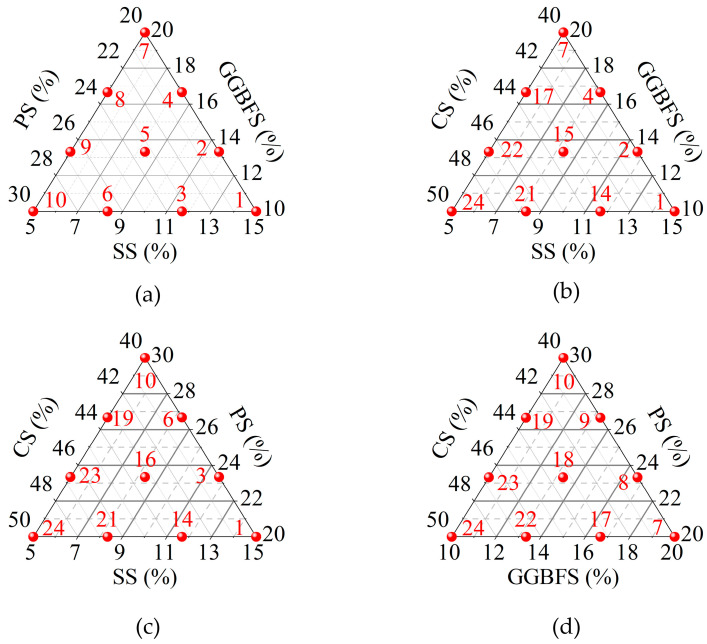
Primary lattices of simplex lattice design: (**a**) CS = 40%; (**b**) PS = 10%; (**c**) GGBFS = 20%; (**d**) SS = 5%.

The basic proportions taken as the center, upper, and lower limits for each ISW were established by allowing for a 10% fluctuation around the center and then making adjustments in terms of constraints and minimum dosages, which served as the boundaries for designing a simplex lattice experiment with three lattice degrees. The primary lattices are shown in Figure 3. The results obtained are described using ternary component response surfaces. Specifically, one factor was fixed, and multiple ternary component response surfaces were employed to illustrate how the response value varies with the other three factors when the fixed factor was held at different levels.

The simplex lattice design was implemented with a degree of 3. Equation (5) was employed to determine the number of primary lattice points, resulting in 20 test points. Additionally, an axial point augmentation design was applied. Given that the number of factors considered was 4, corresponding to 4 axes, 4 axial points were designed. Consequently, the simplex lattice method yielded a total of 24 test points. Including the baseline mixture ratio, a grand total of 25 test points was obtained.

(5) $$n=C_{p+d-1}^{d}$$

where *p* and *d* stand for the number of factors and the degree of the lattice in simplex lattice design.

The proportions and corresponding TCM for each experiment points are provided in Table 1. The mass ratio of CM, standard sand, and water was determined to be 1:3:0.5 according to GB/T 17671-2021 [60], with a curing time of 28 d.

#### 3.2.2. Stabilization of Aeolian Sand Using Developed ISWs-CM

**Figure 4:**
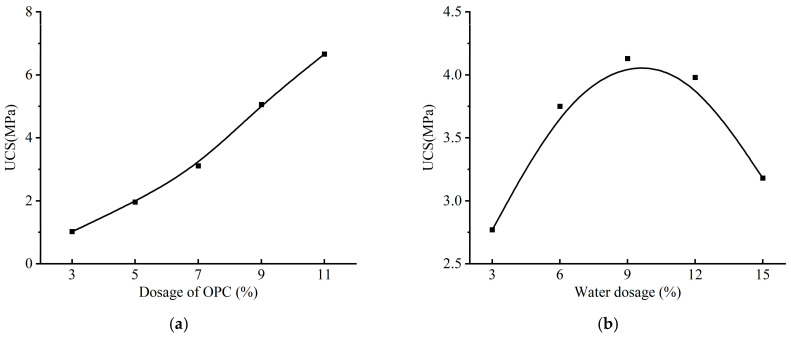
Variation in UCS of ASOPC with cement and water dosages: (**a**) OPC dosage and (**b**) water dosage.

Aeolian sand stabilized with the optimal ISW-CM (ASISW) was compared with that stabilized with OPC (ASOPC) to evaluate the substitutability of the developed ISW-CM for OPC in this application. In previous research [49], an orthogonal experiment was conducted to determine the effects of cement and water dosages on the UCS of ASOPC, with the results presented in Figure 4. Regarding water dosage, it was observed that the strength of ASOPC peaked at a water dosage of 9%; further increases in water dosage led to a decline in strength. Consequently, to enable a direct comparison of the stabilization effects of ISWs-CM and OPC on aeolian sand under identical conditions, the ASISW specimens prepared in this study also incorporated a water dosage equivalent to 9% of the dry sand mass.

Furthermore, Figure 4b indicates that the UCS of ASOPC increased almost linearly with higher cement dosage. However, based on existing research and engineering practice [51], an 11% cement content (relative to dry sand mass) is commonly adopted for stabilizing aeolian sand. In this study, employing an excessively high ISWs-CM dosage could diminish the practical relevance of the findings for engineering applications. Therefore, for the strength testing of ASISW, the ISWs-CM dosage was set at 11% of the dry sand mass. All specimens were cured in a constant temperature and humidity chamber maintained at 23 °C ± 2 °C with relative humidity ≥ 99% for 28 days, which represents the standard duration for evaluating stabilization effectiveness.

### 3.3. Specimen Preparation

As shown in Figure 1, the mortar specimens used in the experiments for developing ISWs-CM were prepared according to the specifications outlined in GB/T 17671-2021 [60]. The mortar prepared comprised 1350 g of standard sand, 450 g of total ISWs, and 225 g of water, which yields a water-to-binder ratio of 0.50 and an ISW-to-sand mass ratio of 1: 3. Dried ISW was initially added to the bowl of an electronic planetary mixer. Low-speed mixing was conducted until a uniform color was achieved, followed by the addition of water and mixing at a low speed for another 30 s. Within the subsequent 30 s, standard sand was slowly introduced. High-speed mixing was performed in a cycle of 30 s of mixing, followed by a 90 s pause and then 90 s of mixing. The mixed mortar was poured into triple 40 mm × 40 mm × 160 mm standard cuboid molds made of polyethylene plastic. The molds were vibrated using a vibrating table to compact the mortar. After 3 days of curing, the molds were removed, and the specimens were placed in water at 20 °C for continued curing.

The cylindrical specimens used in the experiments to investigate the stabilization effect of ISWs-CM on aeolian sand were prepared in accordance with ASTM D5102-2017 [61]. ISWs and aeolian sand were added into the mixer and blended slowly until a uniform color was achieved. Subsequently, water was slowly added into the bowl while the mixer was running. Mixing continued until the color became uniform again, after which high-speed mixing was performed for an additional 5 min. The well-mixed compound was then divided into three layers and loaded into a cylindrical stainless steel mold with a diameter of 50 mm and a height of 100 mm. After each layer was loaded, it was compacted from the outer edge to the center with a tamping rod using 15 strikes, and the surface was roughened with a knife to ensure good bonding with the next layer. After being cured for three days, the mold was removed, and the specimens were placed in a curing chamber to continue curing at 23 °C.

Three repeated experiments were performed for each proportion to minimize error, and UCS was taken as the average of the three specimens.

After the ASISW specimens were crushed, fragments from the internal section were collected. These fragments were ground into powder and subsequently freeze-dried to arrest further hydration reactions. The resulting powder was used for subsequent phase composition and microstructural analyses.

### 3.4. UCS Measurement

An electronic universal hydraulic testing machine was employed to conduct a UCS test, as shown in Figure 1. For the tests of the mortar specimen, a pressure sensor with a resolution of 1 kN and a range of 300 kN was employed. A fixture equipped with three cylindrical spacers was used for the breaking of cuboid specimens, and another fixture featuring a 40 mm × 40 mm loading plate was employed for the compression of the post-break specimens. The cured cuboid specimens were first fractured, and then a load was applied to one half of each specimen at a rate of 2400 N/s until failure occurred.

A sensor with a resolution of 0.1 kN and a range of 10 kN was used to measure the UCS of stabilized aeolian sand specimens. The cured specimens were placed on the loading bottom plate and centered using marks on the bottom plate to avoid eccentric loading conditions. Displacement loading was applied at a rate of 0.5 mm/min until failure occurred. A portion of the destroyed specimen was milled, freeze-dried, and then placed in a vacuum bag for further testing.

The displacement of the specimens during experiments was measured using displacement sensors with a resolution of 0.01 mm.

### 3.5. Mineral Phase Composition

A D8-Advance X-ray diffractometer (Bruker, Hongkong, China) with Cu Kα radiation generated at 60 kV and 80 mA was employed to measure the mineral phase composition of the specimens. The scanning speed was set at 30 steps/s, with a step size of 0.02°, and the scanning range was from 10° to 90°.

### 3.6. Microstructure

An SU8220 field emission scanning electron microscope (HITACHI High-tech Co., Ibaraki, Japan) was utilized to observe the microstructure. The specimens were coated with gold using an ion sputter coater at a current of 10 μA for 2 min, then observed with an electron beam at 5 kV. Energy-dispersive spectroscopy (EDS) was employed to analyze the chemical composition of the particle surface.

### 3.7. Hydration Products

An SDT Q600 synchronous thermal analyzer (TA Instruments Inc., New Castle, DE, USA) was employed to conduct thermogravimetric analysis (TGA) on the stabilized aeolian sand, with the aim of determining the hydration products and their thermal stability. A total of 10 mg of freeze-dried and ground, stabilized aeolian sand was heated at a rate of 10 °C/min in a nitrogen atmosphere from room temperature to 800 °C. The weight–temperature (W-T) curve was recorded during the heating process.

## 4. Results and Analysis

### 4.1. Development of ISWs-CM

**Figure 5:**
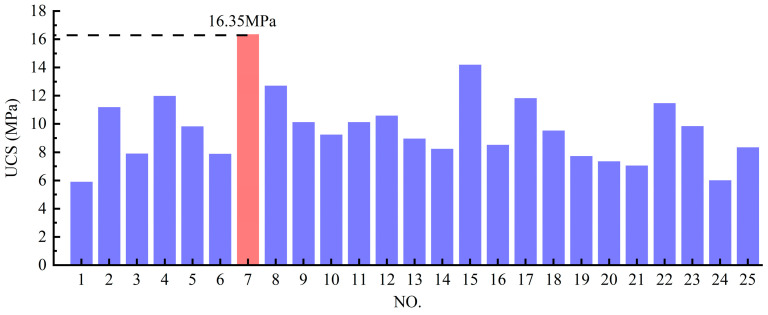
Comparison of specimen UCS in simplex lattice design experiment.

**Figure 6:**
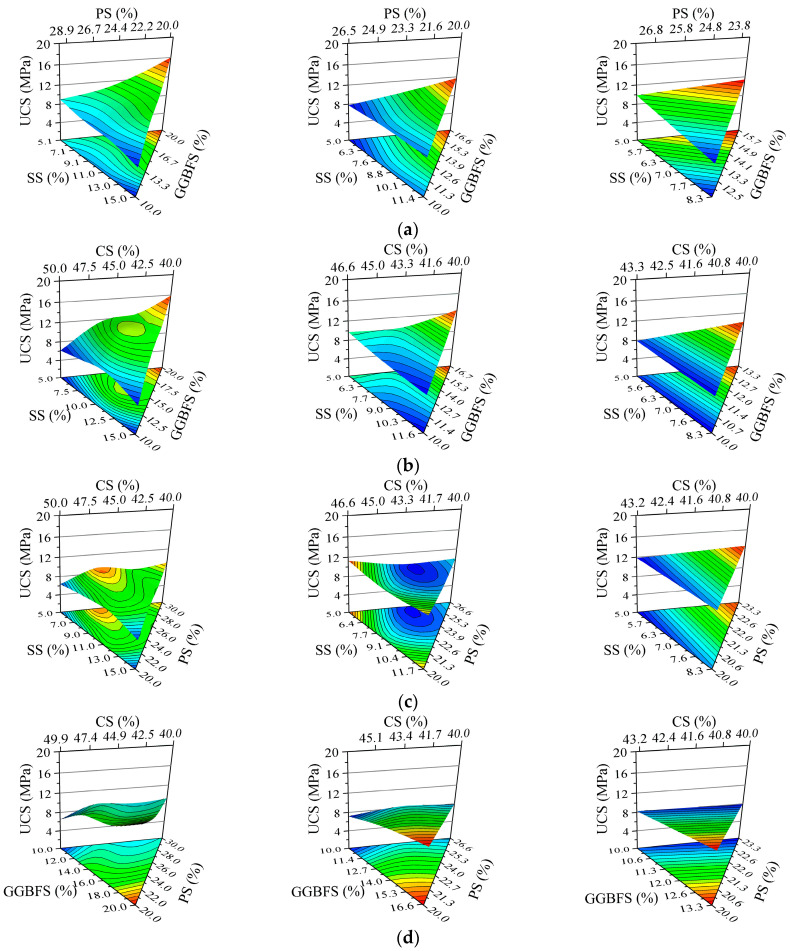
Variation in mortar UCS with proportion of ISWs, where fixed factor is (**a**) CS, (**b**) PS, (**c**) GGBFS, and (**d**) SS.

Table 2 summarizes the UCS test results and corresponding ISW mix proportions for all specimens in the simplex lattice design experiment, while Figure 5 presents a cross-comparison of specimen UCS. The ISW mortar achieved a peak UCS of 16.35 MPa with the solid waste ratio SS:GGBFS:PS:CS = 5:20:20:40 (marked in red), whereas the minimum strength of 5.90 MPa corresponded to SS:GGBFS:PS:CS = 15:10:20:40. It is evident that the specimens with the highest GGBFS content yielded the maximum UCS. Conversely, elevated SS content significantly reduced mortar strength. However, considering potential complex interactions among ISW constituents, the ternary component response surfaces shown in Figure 6 were employed to further analyze the influence of ISW mix proportions on mortar UCS from multiple perspectives. Regions approaching red on the response surface signify higher values, whereas those approaching blue indicate lower values. The ternary component response surfaces are classified according to the regions shown in Figure 3, with the names of ISWs representing dosages.

The fixed factor in Figure 6a is CS. The UCS of mortar increases with increasing GGBFS content and decreases with an increase in SS and PS, regardless of CS dosage. However, when CS is at 40%, the contour lines in the ternary diagram change relatively smoothly, with the UCS of most samples falling within a medium range (around 11 MPa). As CS increases, the rate of change in UCS gradually increases, and the influence of each ISW becomes more pronounced. When CS reaches 46%, the contour lines are nearly perpendicular to the GGBFS axis, indicating that the impact of GGBFS on the UCS of the mortar can be enhanced by an increase in CS. The primary role of CS in the system is to supplement CaO, thereby enhancing alkalinity. A lower CS content results in a low-alkalinity system, which has a relatively minor effect on hydration reactants such as C_2_S. Consequently, it is difficult to discern differences among ISWs with varying reactivity, and significant changes in UCS are only observed when other ISWs approach their limits. As the CS content increases, the differences in reactivity among ISWs become more pronounced in a high-alkalinity environment.

The fixed factor in Figure 6b is PS. Similar trends can be observed compared to when CS is held constant. An increase in GGBFS, along with decreases in SS and CS, leads to an enhancement in the UCS of mortar. The influence of GGBFS becomes more pronounced when PS is increased to 26%. This can be attributed to the similar CaO content in PS and CS, which enables PS to contribute to the alkalinity of the system. Another function of PS is to adjust the ratio of Si-phase to Al-phase oxides, but its hydration reactivity is lower than that of GGBFS. Consequently, when the dosage of PS is high, increasing CS decreases the content of GGBFS—the most reactive hydraulic component—in the system, which reduces the total volume of synthesized hydration products, weakening mechanical performance. Simultaneously, excessive CaO from CS may generate Ca(OH)_2_ that raises the pH of the system to a value beyond the optimal range for hydration product synthesis [62,63,64].

The fixed factor in Figure 6c is GGBFS. Increasing CS first raises mortar UCS, which peaks at 46% CS and then declines. This trend aligns with previous analyses, indicating that although the system requires CS to increase alkalinity, an excessively high proportion can lead to a decrease in the content of hydration-active minerals, thereby reducing strength. When the GGBFS content is increased to 13.3%, the narrowing range of CS and the increased alkalinity demand result in a monotonic increase in the UCS of the mortar with increasing CS. However, when the GGBFS dosage is further increased to 16.6%, the range of UCS variation in the mortar is only 0.9 MPa, suggesting that the mortar strength becomes less susceptible to the influence of other ISWs.

SS serves as the fixed factor in Figure 6d. The UCS of the mortar increases with an increase in GGBFS and decreases with an increase in PS, regardless of the SS dosage. Meanwhile, when CS is increased, the UCS of mortar with an SS dosage not exceeding 8.3% initially rises and then falls but is almost unaffected by CS when the SS dosage is increased to 11.6%. This can be explained by the restricted variation range of CS due to the elevated proportion of SS, as well as the insensitivity to changes in the alkalinity of SS caused by its low hydration reactivity.

The XRD pattern of ISWs presented in Figure 2 reveals that within the scanned range, GGBFS exhibits no clear diffraction peaks and displays a broad peak at approximately 2θ = 20°, indicating its relatively low crystallinity and predominantly amorphous structure, which facilitates its involvement in hydration reactions, making GGBFS the most reactive of the three ISWs used as raw materials for hydration reactions, followed by PS, while SS exhibits the lowest reactivity. This observation leads to a general trend to be seen in the test results: the UCS of mortar increases with an increase in GGBFS dosage.

The relationship between the proportions of ISWs and the UCS of mortars (UCS_m_) was fitted using the polynomial shown in Equation (5). This equation, based on the quartic Scheffé polynomial, was derived by iteratively eliminating statistically insignificant terms. It achieved a goodness-of-fit *R*^2^ of 85.3% and passed the test for the independence, randomness, and normality of residuals.

(6) $${\mathrm{UCS}}_{m}=29x_{1}+1.8x_{2}-7.8x_{3}-0.5x_{4}-256x_{1}x_{2}-61x_{1}x_{4}+59x_{2}x_{3}+18x_{3}x_{4}+545x_{1}x_{2}x_{4}-135x_{2}x_{3}x_{4}$$

where *x*_1_, *x*_2_, *x*_3_, and *x*_4_ stand for the dosages of SS, GGBFS, PS, and CS, respectively, and terms with minor influence are omitted for model simplification. The ANOVA results presented in Table 3 were used to determine the statistical significance of the effects of the considered factors (including interactions between ISWs) on the UCS of the mortar. Due to the intrinsic constraint inherent in mixture design experiments—where altering the proportion of one component necessitates compensatory changes in one or more other components (as their sum must remain constant)—the table does not provide individual *p*-values for individual linear terms. Instead, it reports the collective statistical significance of the overall linear terms (representing the combined effect of all single components) on the UCS. The results of ANOVA indicate that, when the significance level *ɑ* is set at 0.05, there exist significant interactions between SS and GGBFS, as well as between SS, GGBFS, and CS. Specifically, the coefficient of the interaction term of SS and GGBFS is –256, suggesting that their combined effect is less than the simple sum of their individual effects, indicating an antagonistic relationship. This antagonism arises from the much lower reactivity of SS compared to GGBFS. When they coexist in the same system, the presence of SS reduces the proportion of GGBFS, consequently decreasing the proportion of reactive oxides in the system. Meanwhile, the coefficient of the interaction term among SS, GGBFS, and CS is 545, indicating a synergistic effect where their combined effect exceeds the simple sum of their individual effects. This implies that, despite the high reactivity of GGBFS, a certain amount of CS is still necessary to provide an alkaline environment for hydration reactions.

Considering the potential impact on the workability and stability of the cementitious material, it is crucial to maintain an alkalinity and oxide ratio within a reasonable range. Therefore, the optimal proportioning of ISWs, determined through strength prediction and response optimization using the fitted model shown in Equation (6) within the considered boundaries, is finally established as SS:GGBFS:PS:CS = 5:20:20:40 (by mass), supplemented with 15% DG to improve density. Furthermore, the UCS of the basic proportion determined by the standard cement TCM is 50% lower than that of the optimal proportion, which indicates that the cementitious materials obtained using a dual-layer optimization strategy have a higher strength than those only using TCM theory.

### 4.2. Application of Developed ISWs-CM in Aeolian Sand Stabilization

**Figure 7:**
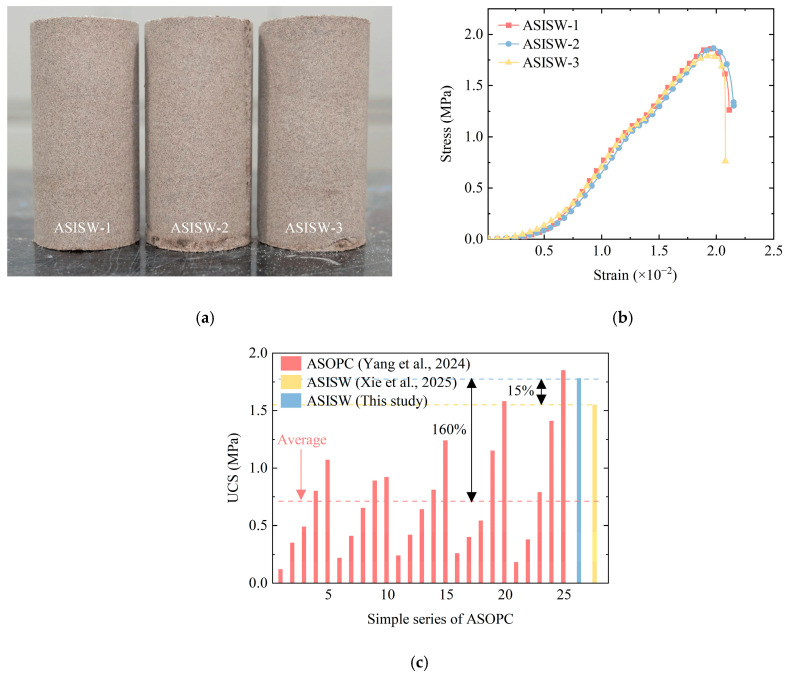
Aeolian sand stabilized by developed ISWs-CM: (**a**) specimens, (**b**) stress–strain curve of ASISW, and (**c**) comparison of UCS of ASISW and ASOPC [44,49].

The ASISW specimens are shown in Figure 7a, where specimen identification numbers for the three parallel tests are labeled. Figure 7b presents the stress–strain curves obtained from UCS tests on these specimens. The curves exhibit three distinct phases: Phase 1, the compaction stage with a progressively increasing slope, during which inter-particle pores gradually close, and particles undergo rearrangement until the maximum density is achieved and stiffness is enhanced; Phase 2, characterized by a stable slope after the completion of pore closure; and Phase 3, where particle-to-particle bonds fracture under compressive stress, initiating and propagating micro-cracks that cause curve descent and specimen failure. The peak stress on the curve corresponds to the specimen’s UCS.

Figure 7c compares the UCS of ASISW prepared in this study with that of ASISW from Xie et al. [44] and ASOPC from Yang et al. [54] The comparison reveals that the average strength of ASOPC is 0.71 MPa, while the specimens prepared at the optimum moisture content (9%) and cured for 28 days reach 0.75 MPa. The UCS of the ASISW prepared by Xie et al. [44] is 1.55 MPa. In contrast, the aeolian sand stabilized with the ISWs-CM developed in this study achieves an average strength of 1.78 MPa—representing increases of 160%, 137%, and 15% over the average ASOPC strength, the ASOPC strength at the equivalent moisture content, and the strength of ASISW prepared by Xie et al. [44], respectively. Although the UCS of ASOPC varies due to different compositions and curing ages, the comparative analysis confirms the potential of the developed ISWs-CM for engineering applications as a cement substitute. These results also demonstrate the positive effect of the additional 15% DG in enhancing the performance of ISWs-CM.

**Figure 8:**
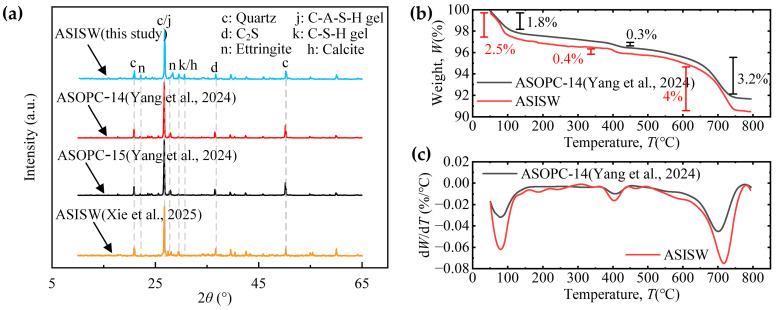
Phase analysis results of ASISW and ASOPC: (**a**) XRD pattern, (**b**) TG curve, and (**c**) DTG curve [44,49].

Figure 8a presents the XRD patterns of ASISW and ASOPC. It can be observed that the gels produced by the hydration of ISWs-CM are primarily C-S-H and C-A-S-H. Characteristic peaks were identified by matching against the PDF4-2009 standard database to determine their corresponding mineral phases. The peak intensities of these two gels in ASISW are higher than those in the two ASOPC samples, and characteristic peaks corresponding to C-S-H or calcite at 2*θ* = 29.5° and 30.6° [8,22,65], which are absent in ASOPC, are observed in ASISW. These observations indicate that ISWs-CM generates more hydrated products in terms of both quantity and variety compared to OPC. Additionally, a comparison of the XRD patterns of aeolian sand stabilized with the ISWs-CM developed in this study and that prepared by Xie et al. [44] reveals significantly stronger diffraction peaks for ettringite (AFt) in the ASISW produced herein. For such highly crystalline phases, an increase in peak intensity typically suggests a relative increase in the content of the corresponding product. This enhancement is attributed to the additional DG, which introduces CaSO_4_·2H_2_O that supplies sufficient precursors for AFt formation, thereby facilitating its synthesis within the system, as shown in Equation (5).

Figure 8b displays a TG curve, while Figure 8c shows differential TG (DTG) curves of ASISW and ASOPC, allowing for the analysis of hydrated products from another perspective through mass loss within different temperature ranges. According to literature reports, the chemically bound water in hydrated products such as AFt and C-S-H gel in inorganic cementitious materials is released between 50 °C and 150 °C, corresponding to the first mass loss stage in Figure 8c and the first peak in the DTG curve. Within this temperature range, the ASISW sample loses 2.5% of its mass, while ASOPC loses 1.8%, indicating a high content of AFt and gel content of ASISW, which is the primary reason for the higher strength observed. The second peak in the DTG curve appears at around 400 °C, with a mass loss of about 0.4% and 0.3% for ASISW and ASOPC in this range, respectively, corresponding to the decomposition of a small amount of remaining Ca(OH)_2_. The last significant mass loss occurs between 600 °C and 750 °C, with a 4% and 3.2% decrease in ASISW and ASOPC samples mass due to the decarbonization decomposition of calcite. The significantly higher calcite content relative to Ca(OH)_2_ in the system can be attributed to the carbonation of Ca(OH)_2_ by CO_2_ during the curing process, coupled with the continuous consumption of OH^−^ and Ca^2+^ during the synthesis of hydration products like C-S-H and AFt, which shifts the dissolution equilibrium of sparingly soluble Ca(OH)_2_ toward dissociation.

This result corroborates the newly emerged calcite characteristic peaks in the XRD pattern. The phase analysis results demonstrate that ISWs-CM can generate more hydrated products and additional crystals that fill particle pores compared to OPC, which is crucial for enhancing the strength of poorly graded aeolian sand.

**Figure 9:**
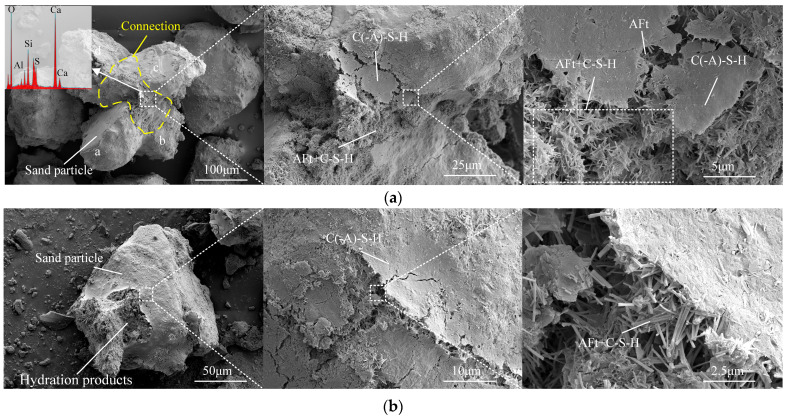
Microscopic morphology of ASISW: (**a**) connection of sand particles and (**b**) sand particle encapsulated by hydration products.

Figure 9a illustrates the typical connection of sand particles in ASISW observed using SEM (SU8220, HITACHI High-tech Co., Ibaraki, Japan). Four sand particles are interconnected by the gel produced through ISWs-CM hydration, forming a relatively stable aggregate. Upon further magnifying the connection, it can be observed that the top layer consists of a lamellar instead of reticulate calcium aluminosilicate hydrate (C(-A)-S-H) gel, which can be confirmed by EDS analysis. Beneath the layer, a large number of rod-like AFt crystals are present, which are bonded together by the gel. This mixed connection of AFt and gel plays a crucial role in filling the inter-particle pores and reinforcing inter-particle bonding. Additionally, the connections between sand particles in ASOPC reported in the literature exhibit numerous apparent pores, resulting in a relatively loose and porous structure, while the connections in ASISW are notably denser. Figure 9b depicts an individual aeolian sand particle within ASISW. This particle is encapsulated by multiple layers of dense hydration products, rendering the surface of aeolian sand barely visible. This observation is analogous to that of particles in ASOPC with high OPC content as reported in the literature. However, in ASOPC particles, the shapes of secondary minerals on the aeolian sand surface are still discernible, suggesting that the hydration product layer may be thinner than that in ASISW. Upon a further magnification of the surface of ASISW particle, a mixed structure of AFt and gel similar to that found at the connection can be observed, which could serve as the basis for the formation of connections between particles.

## 5. Discussion

### 5.1. Substitutability of ISWs-CM for OPC in Engineering Applications

This study employed a dual optimization strategy based on TCM and a simplex lattice design to synergistically prepare a cementitious material from five ISWs. The performance of ASISW was comparable to ASOPC. Specifically, as shown in Figure 7, the 28-day UCS of ASISW reached 1.78 MPa, equivalent to that of ASOPC, demonstrating the substitutability of ISWs-CM for OPC. Additionally, the existing literature indicates that ISWs-CMs have been widely used to stabilize weak soils like clay and silty soil. Although these soils differ mechanically from the aeolian sand in this study, and ISWs-CM dosages vary, quantitative comparisons still validate the effectiveness of the ISWs-CM developed in this work. For instance, Wu et al. [8] reported stabilizing marine soft clay using SS and fly ash (FA), achieving a maximum 28-day UCS of 1.2 MPa—lower than that of ASISW in this study despite the poorer gradation of aeolian sand. In another study by Wu et al. [18] on stabilizing expansive soil with alkali-activated SS, a 10% dosage yielded a 28-day UCS of 1.68 MPa, slightly higher than the ASISW strength. Maneli et al. [20] reported stabilizing black cotton soil with FA, GGBFS, and lime, where the maximum 28-day UCS was 1.0 MPa, significantly lower than the strength of ASISW. Furthermore, the UCS of the ASISW developed in this study meets the strength requirement (1.5–2.5 MPa) for stabilized soil in the sub-base layers of first-class roads and highways in the Chinese Road Specifications (JTG/T F20-2015) [66]. The above quantitative comparisons substantiate the substitutability of the developed ISWs-CM for OPC and its effectiveness for aeolian sand stabilization.

### 5.2. Limitations

Despite the relatively satisfactory results achieved by the ISWs-CM developed in this study for stabilizing aeolian sand, there are still limitations and shortcomings revealed by the experimental data. Firstly, both the ternary diagram analysis results in Figure 6 and the analysis of variance results indicate that among the five ISWs utilized, GGBFS possesses relatively high reactivity, while the reactivity of the other ISWs is low. Consequently, increasing the proportion of GGBFS while decreasing the proportions of other materials can significantly enhance mortar strength. This observation reveals that the majority of materials within the developed ISWs-CM remain in a state of low reaction extent. Treating the ISWs by incorporating chemical activators or employing mechanical activation methods such as high-energy ball milling could significantly enhance their reactivity. This approach would likely increase the reaction extent of the ISWs, thereby synthesizing more hydration products, and potentially serve as an effective method to further improve the strength of ASISW.

On the other hand, for subgrade and foundation engineering projects constructed in desert regions, operation under conditions of high temperature, aridity, and intense UV radiation is inevitable. This study is limited to investigating the mechanical properties of ASISW under standard curing conditions. Understanding how harsh desert conditions affect the strength of ASISW and identifying suitable admixtures to enhance its long-term durability and stability are critically important for the practical implementation of this novel, environmentally friendly ISWs-CM material in desert engineering projects.

## 6. Conclusions

This study optimally designed a universal cementitious material based on five ISWs (including SS, GGBFS, PS, CS, and DG) by fabricating mortar specimens and employing a strategy combining TCM theory and mixture experiments. The optimal proportion of ISWs was determined. Through an application in aeolian sand solidification, substitutability for cement and the high effectiveness of the developed cementitious material were confirmed. The fundamental mechanisms underlying this cement replacement capability were elucidated via phase composition analysis and microstructural characterization. The main conclusions are as follows:

(1) Component reactivity governs ISWs-CM performance, with GGBFS exhibiting dominant hydration activity. Mortar strength increases with GGBFS dosage but decreases with SS content, while CS demonstrates an optimal threshold. The ISW proportion achieving the maximum UCS for mortar is SS:GGBFS:PS:CS = 5:20:20:40. However, this composition has heavy reliance on GGBFS due to the low reactivity of other components, limiting formulation flexibility. Future work should explore activation methods for low-reactivity ISWs.

(2) The ASISW prepared in this study exhibited a 28-day UCS 160% higher than that of ASOPC, and the incorporation of additional DG enhanced the strength of ASISW by 15% compared to a previous study. Compared to soils stabilized by ISWs-CMs reported in the literature, ASISW demonstrated superior strength performance while satisfying the strength requirements for stabilized soils specified in relevant standards. Phase analysis revealed that the hydration products of the developed ISWs-CM were similar to those of OPC but formed in significantly greater quantities. The addition of DG promoted ettringite synthesis, thereby enhancing compactness.

(3) Benefiting from the synergistic effect of the five types of ISWs, aeolian sand stabilized by ISWs-CM achieves performance comparable to that of OPC, making it a viable alternative to OPC as a stabilizer for aeolian sand. The developed cementitious material exhibits potential to act as a substitute for OPC in stabilizing a wider range of problematic soils and other engineering applications conducted as a mortar-based experiment.

(4) The long-term performance of the developed ISWs-CM under harsh desert environments—characterized by elevated temperatures and intense UV radiation—requires targeted durability investigations. The workability properties of ISWs-CM, including fluidity and setting time, as well as its field applications in desert environments, represent potential research directions for future investigations.

## Figures and Tables

**Table 1:** Proportion and UCS of specimens in simplex lattice design.

NO.	Proportion (%)					TCM		
	SS	GGBFS	PS	CS	DG	SM	IM	KH
1	15.00	10.00	20.00	40.00	15	1.61	0.79	0.89
2	11.67	13.33	20.00	40.00		1.78	1.07	0.85
3	11.67	10.00	23.33	40.00		1.91	0.94	0.87
4	8.33	16.67	20.00	40.00		1.98	1.51	0.82
5	8.33	13.33	23.33	40.00		2.13	1.35	0.84
6	8.33	10.00	26.67	40.00		2.31	1.19	0.86
7	5.00	20.00	20.00	40.00		2.20	2.29	0.80
8	5.00	16.67	23.33	40.00		2.38	2.08	0.81
9	5.00	13.33	26.67	40.00		2.59	1.86	0.83
10	5.00	10.00	30.00	40.00		2.85	1.64	0.84
11	11.25	11.25	21.25	41.25		1.85	1.00	0.90
12	6.25	16.25	21.25	41.25		2.18	1.77	0.85
13	6.25	11.25	26.25	41.25		2.47	1.48	0.87
14	11.67	10.00	20.00	43.33		1.78	0.91	0.97
15	8.33	13.33	20.00	43.33		1.99	1.30	0.93
16	8.33	10.00	23.33	43.33		2.15	1.14	0.95
17	5.00	16.67	20.00	43.33		2.22	2.00	0.90
18	5.00	13.33	23.33	43.33		2.43	1.79	0.92
19	5.00	10.00	26.67	43.33		2.67	1.57	0.93
20	6.25	11.25	21.25	46.25		2.22	1.40	1.02
21	8.33	10.00	20.00	46.67		2.00	1.10	1.06
22	5.00	13.33	20.00	46.67		2.26	1.72	1.02
23	5.00	10.00	23.33	46.67		2.48	1.50	1.04
24	5.00	10.00	20.00	50.00		2.30	1.44	1.16
25	7.30	13.71	19.42	44.72		2.03	1.42	0.97

**Table 2:** UCS of specimens in simplex lattice design experiment.

NO.	Proportion (%)					UCS (MPa)
	SS	GGBFS	PS	CS	DG	
1	15.00	10.00	20.00	40.00	15	5.90
2	11.67	13.33	20.00	40.00		11.20
3	11.67	10.00	23.33	40.00		7.91
4	8.33	16.67	20.00	40.00		11.98
5	8.33	13.33	23.33	40.00		9.83
6	8.33	10.00	26.67	40.00		7.88
7	5.00	20.00	20.00	40.00		16.35
8	5.00	16.67	23.33	40.00		12.71
9	5.00	13.33	26.67	40.00		10.13
10	5.00	10.00	30.00	40.00		9.24
11	11.25	11.25	21.25	41.25		10.13
12	6.25	16.25	21.25	41.25		10.60
13	6.25	11.25	26.25	41.25		8.96
14	11.67	10.00	20.00	43.33		8.24
15	8.33	13.33	20.00	43.33		14.20
16	8.33	10.00	23.33	43.33		8.53
17	5.00	16.67	20.00	43.33		11.83
18	5.00	13.33	23.33	43.33		9.53
19	5.00	10.00	26.67	43.33		7.72
20	6.25	11.25	21.25	46.25		7.36
21	8.33	10.00	20.00	46.67		7.05
22	5.00	13.33	20.00	46.67		11.47
23	5.00	10.00	23.33	46.67		9.85
24	5.00	10.00	20.00	50.00		6.01
25	7.30	13.71	19.42	44.72		8.35

**Table 3:** Results of ANOVA.

Source		Degree of Freedom, *DOF*	Sequential Sums of Squares, Seq *S*	Sequential Mean Squares, Seq *MS*	*p*-Value
Linear		3	103.18	4.62	0.083
Quadratic	SS×GGBFS	1	1.423	8.835	0.040
	SS×CS	1	2.888	7.536	0.055
	GGBFS×PS	1	3.356	6.427	0.073
	PS×CS	1	0.331	7.892	0.050
Cubic	SS×GGBFS×CCR	1	2.353	9.082	0.037
	GGBFS×PS×CCR	1	7.462	7.462	0.056
Residual error		14	24.008	1.715	
Total		23	145.010		

## Data Availability

The raw data supporting the conclusions of this article will be made available by the authors upon request.

## Abbreviations

ISW	industrial solid waste
ISWs-CM	industrial solid wastes based cementitious materials
SS	steel slag
GGBFS	ground granulated blast-furnace slag
PS	phosphorus slag
CS	carbide slag
DG	desulfurized gypsum
OPC	ordinary Portland cement
TCM	three chemical moduli
IM	alumina modulus
SM	silica modulus
KH	lime saturation coefficient
C-S-H	calcium silicate hydrate
UCS	unconfined compression strength
C2S	dicalcium silicate
C3S	tricalcium silicate
CSS	coarse steel slag
CPS	coarse phosphorus slag
CCS	coarse carbide slag
SEM	scanning electron microscopy
EDS	energy dispersive spectroscopy
XRD	X-ray diffraction
XRF	X-ray fluorescence
TGA	thermogravimetric analysis
DTG	differential thermogravimetric analysis
ASISW	aeolian sand stabilized by industrial solid wastes based cementitious materials
ASOPC	aeolian sand stabilized by ordinary Portland cement
AFt	ettringite
ANOVA	analysis of variance

## References

[1] Lorenzo G.A., Bergado D.T. (2004). Fundamental parameters of cement-admixed clay—New approach. J. Geotech. Geoenviron. Eng..

[2] Wu J., Deng Y.F., Zheng X.P., Cui Y.J., Zhao Z.P., Chen Y.G., Zha F.S. (2019). Hydraulic conductivity and strength of foamed cement-stabilized marine clay. Constr. Build. Mater..

[3] Wu J., Liu L., Deng Y.F., Zhang G.P., Zhou A.N., Wang Q. (2021). Distinguishing the effects of cementation versus density on the mechanical behavior of cement-based stabilized clays. Constr. Build. Mater..

[4] Wu J., Liu L., Deng Y.F., Zhang G.P., Zhou A.N., Xiao H.L. (2021). Use of recycled gypsum in the cement-based stabilization of very soft clays and its micro-mechanism. J. Rock Mech. Geotech. Eng..

[5] Xu M., Liu L., Deng Y.F., Zhou A.N., Gu S.T., Ding J.W. (2021). Influence of sand incorporation on unconfined compression strength of cement-based stabilized soft clay. Soils Found..

[6] Cunningham P.R., Wang L., Nassiri S., Thy P., Harvey J.T., Jenkins B.M., Miller S.A. (2025). Compressive strength and regional supply implications of rice straw and rice hull ashes used as supplementary cementitious materials. Resour. Conserv. Recycl..

[7] Navaratnam S., Tushar Q., Jahan I., Zhang G.M. (2023). Environmental Sustainability of Industrial Waste-Based Cementitious Materials: A Review, Experimental Investigation and Life-Cycle Assessment. Sustainability.

[8] Wu J., Deng Y.F., Zhang G.P., Zhou A.N., Tan Y.Z., Xiao H.L., Zheng Q.S. (2021). A Generic Framework of Unifying Industrial By-products for Soil Stabilization. J. Clean. Prod..

[9] Duan D.D., Wu H.B., Wei F., Song H.P., Chen Z., Cheng F.Q. (2023). Preparation, characterization, and rheological analysis of eco-friendly geopolymer grouting cementitious materials based on industrial solid wastes. J. Build. Eng..

[10] Ke Y., Chen Y., Liang S., Hu J.P., Hou H.J., Quan J.D., Li X.W., Duan H.B., Yuan S.S., Yang J.K. (2023). Environmentally sound management of industrial solid waste: A paradigm of proposed bi-tetrahedron. Resour. Conserv. Recycl..

[11] Kumar N., Amritphale S.S., Matthews J.C., Lynam J.G., Alam S., Omar A.A. (2021). Synergistic utilization of diverse industrial wastes for reutilization in steel production and their geopolymerization potential. Waste Manag..

[12] Li Z.P., Zhang J.Y., Lei Z.X., Gao M.S., Sun J.B., Tong L.H., Chen S.M., Wang Y.F. (2024). Designing low-carbon fly ash based geopolymer with red mud and blast furnace slag wastes: Performance, microstructure and mechanism. J. Environ. Manag..

[13] Lu X.L., Yu Q., Xu J.Y., Yue B., Sheng M.Q. (2023). Comparative Experimental Study on Strength Properties of Red Clay Modified by Cement and Industrial Solid Waste Powder. Adv. Civ. Eng..

[14] Wang X.L., Wang X.C., Fu P.F., Shi J.J., Xu M. (2024). Performance Optimization of Alkaline Multi-Industrial Waste-Based Cementitious Materials for Soil Solidification. Materials.

[15] Zhang Z., Guan C., Hua S.D., Zhang Y.N., Zhang D.R., Bao Y.Z., Yuan Z.Z. (2024). Performance Evaluation and Mechanism Study of Solid Waste-Based Cementitious Materials for Solidifying Marine Soft Soil under Seawater Mixing and Erosion Action. Appl. Sci..

[16] Duan S.Y., Liao H.Q., Cheng F.Q., Song H.P., Yang H.Q. (2018). Investigation into the synergistic effects in hydrated gelling systems containing fly ash, desulfurization gypsum and steel slag. Constr. Build. Mater..

[17] Gu X.Y., Yu B., Dong Q., Deng Y.F. (2018). Application of secondary steel slag in subgrade: Performance evaluation and enhancement. J. Clean. Prod..

[18] Wu J., Liu Q.W., Deng Y.F., Yu X.B., Feng Q., Yan C. (2019). Expansive soil modified by waste steel slag and its application in subbase layer of highways. Soils Found..

[19] Yang P., Liu L., Suo Y.L., Qu H.S., Xie G., Zhang C.X., Deng S.C., Lv Y. (2023). Investigating the synergistic effects of magnesia-coal slag based solid waste cementitious materials and its basic characteristics as a backfill material. Sci. Total Environ..

[20] Maneli A., Kupolati W.K., Abiola O.S., Ndambuki J.M. (2016). Influence of fly ash, ground-granulated blast furnace slag and lime on unconfined compressive strength of black cotton soil. Road Mater. Pavement Des..

[21] Yi Y.L., Liska M., Jin F., Al-Tabbaa A. (2016). Mechanism of reactive magnesia—ground granulated blastfurnace slag (GGBS) soil stabilization. Can. Geotech. J..

[22] Liu J.P., Song G., Ge X.W., Liu B., Liu K.X., Tian Y.L., Wang X., Hu Z.H. (2024). Experimental Study on the Properties and Hydration Mechanism of Gypsum-Based Composite Cementitious Materials. Buildings.

[23] Wu P.F., Liu X.Y., Liu X.M., Zhang Z.Q., Wei C. (2024). Effect of Industrial Byproduct Gypsum on the Mechanical Properties and Stabilization of Hazardous Elements of Cementitious Materials: A Review. Materials.

[24] Chen T.F., Gao Y.L., Li Y.L., Zhu J.C., Cheng Z.D., Xiong H.Y. (2024). The strength, reaction mechanism, sustainable potential of full solid waste alkali-activated cementitious materials using red mud and carbide slag. Constr. Build. Mater..

[25] Li Y.C., Min X.B., Ke Y., Liu D.G., Tang C.J. (2019). Preparation of red mud-based geopolymer materials from MSWI fly ash and red mud by mechanical activation. Waste Manag..

[26] Li Z.F., Zhang J., Li S.C., Lin C.J., Gao Y.F., Liu C. (2021). Feasibility of preparing red mud-based cementitious materials: Synergistic utilization of industrial solid waste, waste heat, and tail gas. J. Clean. Prod..

[27] Wang J., Liu X.M., Zhang Z.Q., Liu Y. (2024). Synergistic utilization, critical mechanisms, and environmental suitability of bauxite residue (red mud) based multi-solid wastes cementitious materials and special concrete. J. Environ. Manag..

[28] Wang Y.G., Liu X.M., Zhu X., Zhu W.X., Yue J.W. (2023). Synergistic effect of red mud, desulfurized gypsum and fly ash in cementitious materials: Mechanical performances and microstructure. Constr. Build. Mater..

[29] Adesina A. (2025). Synthesis, characterization, and efficacy of alkali-activated materials from mine tailings: A review. Waste Manag..

[30] Simonsen A.M.T., Solismaa S., Hansen H.K., Jensen P.E. (2020). Evaluation of mine tailings’ potential as supplementary cementitious materials based on chemical, mineralogical and physical characteristics. Waste Manag..

[31] Waleed M., Alshawmar F. (2025). Enhancing mechanical properties of low plasticity soil through coal and silica fume stabilization. Sci. Rep..

[32] Umar I.H., Lin H., Ibrahim A.S. (2023). Laboratory Testing and Analysis of Clay Soil Stabilization Using Waste Marble Powder. Appl. Sci..

[33] Cai D.G., Ouyang M.Z., Bao X.Y., Zhang Q.L., Bi Z.Q., Yan H.Y., Li S.M., Shi Y.F. (2025). Performance Evaluation of Stabilized Soils with Selected Common Waste Materials of Rice Husk Ash, Steel Slag and Iron Tailing Powder. Materials.

[34] Pastor J.L., Chai J.C., Sánchez I. (2023). Strength and Microstructure of a Clayey Soil Stabilized with Natural Stone Industry Waste and Lime or Cement. Appl. Sci..

[35] Gücek S., Gürer C., Žlender B., Taciroğlu M.V., Korkmaz B.E., Gürkan K., Bračko T., Macuh B., Varga R., Jelušič P. (2024). Use of Lignin, Waste Tire Rubber, and Waste Glass for Soil Stabilization. Appl. Sci..

[36] Tanyildizi M., Uz V.E., Gökalp I. (2023). Utilization of waste materials in the stabilization of expansive pavement subgrade: An extensive review. Constr. Build. Mater..

[37] Ikeagwuani C.C., Nwonu D.C. (2022). Application of fuzzy logic and grey based Taguchi approach for additives optimization in expansive soil treatment. Road Mater. Pavement Des..

[38] Tang P.P., Javadi A.A., Vinai R. (2025). Calcium carbide residue for clay stabilisation: Mechanical and microstructural properties. Transp. Geotech..

[39] Ikeagwuani C.C., Alexander T.C., Odumade A.O. (2024). Evaluation of strength development and micro-pore characteristics of stabilized expansive soil. Environ. Earth Sci..

[40] Ikeagwuani C.C., Nwonu D.C., Onah H.N. (2021). Min-max fuzzy goal programming—Taguchi model for multiple additives optimization in expansive soil improvement. Int. J. Numer. Anal. Methods Geomech..

[41] Nwonu D.C., Onyia M.E. (2023). Novel grey-vector optimization of desiccation-induced shrinkage and strength of industrial waste-based soil-composite binder as sustainable construction material. J. Mater. Cycles Waste Manag..

[42] Hamed E., Demiröz A. (2024). Optimization of geotechnical characteristics of clayey soils using fly ash and granulated blast furnace slag-based geopolymer. Constr. Build. Mater..

[43] Ashraf M.S., Ghouleh Z., Shao Y.X. (2019). Production of eco-cement exclusively from municipal solid waste incineration residues. Resour. Conserv. Recycl..

[44] Xie Z.L., Qian Z.Z., Wang H., Qi Y.Z., Yue B. (2025). Synergistic Preparation and Mechanistic Investigation of Full Industrial Solid Waste-Based Cementitious Materials for Aeolian Sand Stabilization. Appl. Sci..

[45] Costa F.N., Ribeiro D.V. (2020). Reduction in CO_2_ emissions during production of cement, with partial replacement of traditional raw materials by civil construction waste (CCW). J. Clean. Prod..

[46] Taylor H.F.W. (1997). Cement Chemistry.

[47] Vilaplana A.S.D., Ferreira V.J., López-Sabirón A.M., Aranda-Usón A., Lausín-González C., Berganza-Conde C., Ferreira G. (2015). Utilization of Ladle Furnace slag from a steelwork for laboratory scale production of Portland cement. Constr. Build. Mater..

[48] Qian Z.Z., Sheng M.Q., Huang F.M., Lu X.L. (2021). Uplift Performance of Plate Anchors in Cement-Stabilised Aeolian Sand. Front. Earth Sci..

[49] Yang H., Qian Z.Z., Yue B., Xie Z.L. (2024). Effects of Cement Dosage, Curing Time, and Water Dosage on the Strength of Cement-Stabilized Aeolian Sand Based on Macroscopic and Microscopic Tests. Materials.

[50] Elipe M.G.M., López-Querol S. (2014). Aeolian sands: Characterization, options of improvement and possible employment in construction—The State-of-the-art. Constr. Build. Mater..

[51] Cui Q., Liu G., Zhang Z.H., Fang Y.Q., Gu X.D. (2023). Experimental Investigation on the Strength and Microscopic Properties of Cement-Stabilized Aeolian Sand. Buildings.

[52] Liu W.Z., Huang X.J., Yin W.H., Liu G.Y. (2025). Static and dynamic characteristics of cement-treated and untreated aeolian sand from the Tengger desert hinterland: Laboratory tests and prediction models. Constr. Build. Mater..

[53] Sun Q., Zhang J.X., Zhou N. (2018). Early-Age Strength of Aeolian Sand-Based Cemented Backfilling Materials: Experimental Results. Arab. J. Sci. Eng..

[54] Yang X., Hu Z.Q., Wang Y., Wang X.L. (2024). Aeolian sand stabilized by using fiber- and silt-reinforced cement: Mechanical properties, microstructure evolution, and reinforcement mechanism. Constr. Build. Mater..

[55] Zhang X.D., Geng J., Pang S., Su L.J., Cai G.J., Zhou Z.C. (2023). Microscopic Properties and Splitting Tensile Strength of Fiber-Modified Cement-Stabilized Aeolian Sand. J. Mater. Civ. Eng..

[56] Liu J., Wang B., Hu C.T., Chen J.G., Zhu S.Y., Xu X.D. (2023). Multiscale study of the road performance of cement and fly ash stabilized aeolian sand gravel base. Constr. Build. Mater..

[57] (2017). Ground Granulated Blast Furnace Slag Used for Cement, Mortar, and Concrete.

[58] (2020). Cement—Test Methods—Determination of Strength.

[59] (2025). Standard Practice for Classification of Soils for Engineering Purposes (Unified Soil Classification System).

[60] (2021). Test Method of Cement Mortar Strength (ISO Method).

[61] (2017). Standard Test Method for Unconfined Compressive Strength of Compacted Soil-Lime Mixtures.

[62] Ba H.J., Li J.J., Ni W., Li Y., Ju Y.J., Zhao B., Wen G.P., Hitch M. (2023). Effect of calcium to silicon ratio on the microstructure of hydrated calcium silicate gels prepared under medium alkalinity. Constr. Build. Mater..

[63] Gong J.W., Zhang K., Xie G.C., Shi K.B., Zhu Y. (2025). Factors Affecting Synthesized C-S-H CO_2_ Uptake: Initial Alkalinity and Ca/Si. Buildings.

[64] Shen X.Y., Feng P., Zhang Q., Lu J.Y., Liu X., Ma Y.F., Jin P., Wang W., Ran Q.P., Hong J.X. (2023). Toward the formation mechanism of synthetic calcium silicate hydrate (C-S-H)- pH and kinetic considerations. Cem. Concr. Res..

[65] Liang S.H., Chen J.T., Guo M.X., Feng D.L., Liu L., Qi T. (2020). Utilization of pretreated municipal solid waste incineration fly ash for cement-stabilized soil. Waste Manag..

[66] (2015). Technical Guidelines for Construction of Highway Roadbases.

